# Tridiet-enhanced weight gain in Sprague Dawley rats: a retrospective analysis

**DOI:** 10.3389/fnut.2026.1740398

**Published:** 2026-02-11

**Authors:** Amanda L. Rodriguez, Cameron Bradfield, Nicholas V. Scarpa, Bianca E. Barroso, Michael I. Fernandez, Sofia V. Cabanillas, Abigail Deleon-Pena, Elise Frias, Victor I. Agboli, Shoumi Sarkar, Somnath Datta, Matthew A. Schiefer

**Affiliations:** 1 Malcom Randall Veterans Affairs Medical Center, North Florida/South Georgia Veterans Health System, Gainesville, FL, United States; 2 Department of Biostatistics, University of Florida, Gainesville, FL, United States; 3 Global Biometrics and Data Sciences, Bristol Myers Squibb, Princeton, NJ, United States; 4 Department of Biomedical Engineering, University of Florida, Gainesville, FL, United States

**Keywords:** diet: high-carbohydrate, diet: high-fat, obesity/physiopathology, rats: Sprague Dawley, weight gain/physiology

## Abstract

**Introduction:**

Obesity research, including our prior studies, commonly utilizes high-fat (HF) diets to induce weight gain in animal models. Here, we report on outcomes during *ad libitum* access to a triple (TRI) diet consisting of HF, high-glucose (HG), and standard (ST) chow diets.

**Methods:**

This retrospective analysis aimed to determine if rats on the TRI diet experienced greater weight gain compared to rats on an HF or ST diet. Previous experimental data from 29 rats were categorized into one of three diet groups: TRI, HF, or ST. Daily food intake and weekly body weights recorded from postnatal days 98 to 182 were analyzed. Caloric intake was calculated based on food consumption and macronutrient composition. Statistical analyses, including confidence intervals and growth modeling, were conducted to assess differences in weight gain patterns across diet groups.

**Results:**

Significant divergence in body weight emerged early, with differences between the TRI and ST groups evident by 7 days on diet and between the TRI and HF groups by 22 days on diet. TRI rats consumed the highest average daily calories, exceeding both other groups.

**Discussion:**

For studies focused on developing obese rodents and without specific dietary restrictions, the TRI diet produces heavier animal models faster, potentially reducing study duration and costs.

## Introduction

Obesity is a global health concern marked by excessive adiposity and associated metabolic dysfunctions (1). Experimental animal models are commonly used to investigate obesity and test potential interventions. Rats are a common model due to physiological similarities to humans, manageable size, and well-characterized metabolic responses (2).

In studies modeling diet-induced obesity (DIO), high-fat (HF) diets are the primary approach due to their capacity to promote weight gain and metabolic disturbances. A review of the literature indicates that most DIO models employ HF diets (3–7); however, HF diets may not be the optimal choice, and other dietary combinations could potentially produce more pronounced effects. Alternative diets, including high-glucose (HG), high-fructose, and combination diets, have been investigated for inducing obesity and can yield different metabolic outcomes (8, 9). Emerging evidence suggests that dietary variety can influence weight gain and energy intake (10). With the idea of variety in mind, this study analyzes the triple (TRI) diet consisting of HF, HG, and standard (ST) chow diets.

As our observations suggested that rats on a TRI diet gained weight more rapidly than those in our previous HF-only studies (10), we conducted a retrospective comparative analysis to determine if the TRI diet led to greater weight gain and increased food consumption. A model that induces more pronounced weight gain could improve experimental efficiency by reducing the time and resources required to study obesity-related outcomes.

## Methods

This retrospective analysis aimed to compare animal weights and food consumption across diet type, duration on diet, and age, and to compare weight gain in Sprague Dawley (SD) rats fed TRI, HF, or ST diet. A total of 29 male SD rats were included in the analysis. Portions of the data included in this retrospective report were presented in a previously published article (10). All rats belonged to control groups from their respective studies and did not receive any additional interventions. They were divided into three diet groups: TRI (*n* = 8), HF (*n* = 12), and ST (*n* = 9).

All rats were individually housed in large acrylic cages measuring approximately 50 cm on each side. They had *ad libitum* access to their assigned diets and water. The TRI group had simultaneous access to three diet types: ST (Teklad Global 18% Protein Rodent Diet 2018), HF (Envigo Teklad TD.06414), and HG (Envigo Teklad TD.08806). The HF group received only the HF chow, while the ST group received only the ST chow.

Body weight was recorded weekly from postnatal day 98 to 182 using a precision digital scale. Food intake was measured on a per animal basis based on weight (g). Food was measured on a daily occurrence (with slight variance), and the difference in food mass observed in the cage was determined to be the grams eaten by the animal. Each rat in the TRI group was provided with three separate bowls containing ST, HF, and HG chow. Each chow type consisted of visually distinct pellets (different colors), which allowed us to easily differentiate between diets and accurately track intake for each type. Rats had continuous *ad libitum* access to all three diet types throughout the study. Although diet-specific gram consumption was recorded, caloric intake was calculated as a simple aggregate: calories from ST, HF, and HG diets were computed separately using the manufacturer’s kcal/g values and then summed to produce each rat’s total daily caloric intake. Since intake was recorded independently for each chow type, we were also able to analyze patterns of diet preference within the TRI group (Supplementary Figure 1). Macronutrient composition was calculated using the same method (Supplementary Figure 2). Details of each diet composition are presented in Table 1.

**Table 1:** Diet composition: energy density and macronutrient distribution across three chow types.

Diet type	Energy density (kcal/g)	% kcal from		
		Protein	Carbohydrate	Fat
ST	3.1	24.0	58.0	18.0
HG	3.6	20.5	69.1	10.5
HF	5.1	18.3	21.4	60.3

### Statistical analysis

To assess the effect of diet type, statistical analyses were conducted to evaluate differences in body weight, food consumption, and caloric intake. Body weight data were standardized by age, and weekly values were interpolated across measured time intervals of 7 ± 5 days (mean ± standard deviation). For the analysis of weight gain, 95% confidence intervals (CIs) were calculated for each group for postnatal days 98–182 (427 observations), which was the period with the greatest overlap in available data across all studies. To validate our ST data, we compared our growth curve to publicly available data from Charles River (CR) (11).

Daily food consumption analyses were limited to postnatal days 98–175 (1,795 observations), as food intake records for some animals were not recorded due to scheduling challenges. This period was selected to ensure complete dietary records and consistent data availability for all groups. Prior to postnatal day 98, rats in the TRI and HF groups had been maintained on their respective diets for 71 ± 3 and 29 ± 7 days (mean ± standard deviation), respectively; all ST rats were maintained on ST chow continuously from weaning until the end of each experimental trial.

To compare different diet types, a one-way analysis of variance (ANOVA) was conducted at each time point to compare mean body weight. ANOVA tests were run for both calorie and gram consumption. Assumptions for the ANOVAs were tested and satisfied, including independence of observations, approximate normality of residuals, as assessed by the Anderson–Darling test (*p* ≥ 0.05), and homogeneity of variances was verified using Levene’s test (*p* ≥ 0.05). The null hypothesis of equal mean body weight across diet groups (H_0_: *α*_1_ = α _2_ = α _3_) was tested at each time point. Where significant group effects were observed, *post-hoc* pairwise comparisons were performed using Tukey’s Honestly Significant Difference (HSD) test.

To analyze non-linear growth trajectories and account for baseline differences in body weight, generalized additive mixed models (GAMMs) were fitted in R (v4.4.1) using the gamm4 package. The model assessed cumulative weight gain as a function of time and diet group. It also incorporated random intercepts and slopes for each rat and diet-specific smooth terms estimated via thin-plate regression splines. Mathematically, the model was given as follows:


$$\Delta Y_{\mathrm{ij}}(t)=Y_{\mathrm{ij}}(t)-Y_{\mathrm{ij}}(t_{0})$$

where $Y_{\mathrm{ij}}(t)\;$ was the observed body weight (g) at time *t* and $Y_{\mathrm{ij}}(t_{0})$ was the baseline (day 0) body weight (g). Body weight terms were subsequently modeled as follows:


$$Y_{\mathrm{ij}}(t)=\beta_{0}+\beta_{i}+f_{i}(t)+b_{\mathrm{oj}}+b_{1j}t+\varepsilon_{\mathrm{ij}}(t)$$

where $\beta_{0}$ was the overall intercept, $\beta_{i}\;$ represented the fixed effect of diet group i , $f_{i}(t)\;$ was the smooth non-linear function of time as a diet i , estimated by thin-plate regression splines. $b_{\mathrm{oj}}$ and $b_{1j}t$ were random intercept and slope for rat j , respectively. The inclusion of random intercepts and slopes allowed each rat to have its own baseline level and rate of growth, while the smooth function $f_{i}(t)$ captured non-linear diet-specific growth patterns over time. The “Days on Diet” variable was used as the standardized time variable to align rats with different diet initiation dates. Rat ID was included as a random effect. Divergence in growth trajectories between diet groups was assessed by examining the overlap of 95% confidence intervals for the fitted smooths. All statistical analyses were performed using R and Minitab (v.22.2.1). The figures were created in MATLAB (R2024a).

### Animals and ethical considerations

All applicable institutional and national guidelines for the care and use of animals were followed throughout the studies. The use of these animals was approved by the Institutional Animal Care and Use Committee (IACUC) at Malcom Randall Veterans Affairs Medical Center (VAMC). All experimental procedures were conducted in accordance with regulations and guidelines established by the Public Health Service, the Animal Welfare Act, the Guide for the Care and Use of Laboratory Animals, and the Animal Research: Reporting of *In Vivo* Experiments (ARRIVE) guidelines. All researchers involved in animal handling and experimentation completed the required training through the Collaborative Institutional Training Initiative (CITI) and the Training Management System (TMS).

## Results

### Weight gain

Body weight gain was assessed from postnatal days 98–182 across the TRI, HF, and ST diet groups. Rats in the TRI group demonstrated the greatest overall increase throughout the timeframe, with a mean gain of 250 ± 59 g. In comparison, the HF group gained 198 ± 33 g (21% less than TRI), while the ST group showed the lowest weight gain at 131 ± 16 g (48% less than TRI).

At postnatal day 98, analysis revealed a statistically significant difference in mean body weight among the TRI, HF, and ST groups (*p* < 0.001, *F* (2, 26) = 40.17). Assumptions for ANOVA and Tukey’s HSD were tested and met, including independence of observations, approximate normality of residuals (Anderson–Darling), and homogeneity of variance (Levene’s test). *Post-hoc* analysis using Tukey’s HSD indicated that rats in the TRI group were significantly heavier than those in the HF and ST groups (*p* < 0.001), while the difference between HF and ST was not statistically significant (*p* = 0.051). Grouping results placed TRI in a distinct category, reflecting its greater weight gain relative to the other diets.

**Figure 1:**
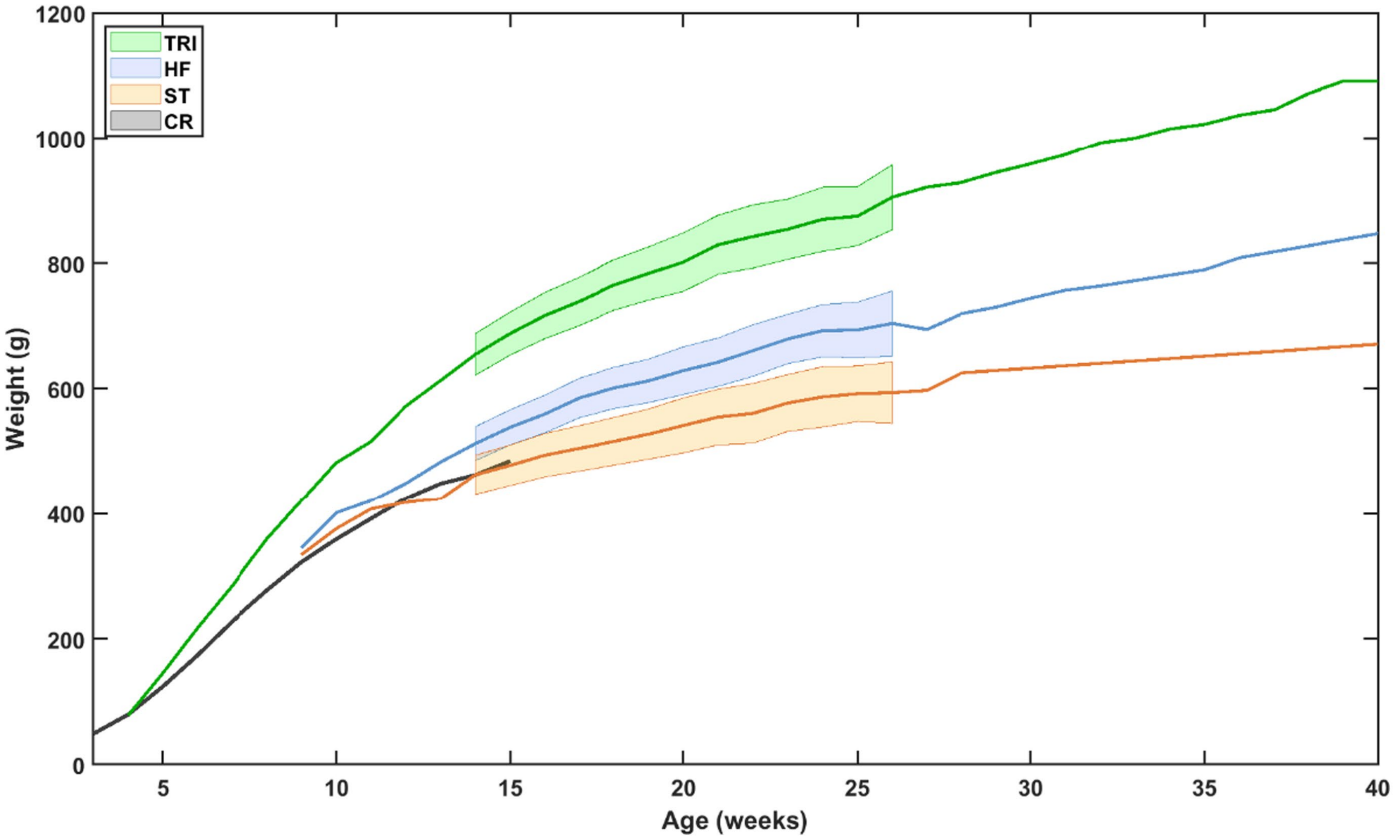
Mean weight as a function of age and diet. For the time period over which statistical tests were run and for which the number of animals in each group is the highest, the 95% confidence interval has also been provided. All weight measurements were aligned by age, and interpolated values were calculated using an average interval of 6.51 ± 5.22 days. Black line: the expected ST weight obtained from publicly available growth charts on the CR website, showing the rats in our study on the ST diet responded as predicted by CR (11).

Body weight increased steadily over time across all groups (Figure 1). Mean body weights on day 98 were 655 ± 61 g for TRI, 513 ± 44 g for HF, and 463 ± 29 g for ST. By day 147, mean weights were 829 ± 96 g for TRI, 642 ± 60 g for HF, and 555 ± 24 g for ST. By the final measurement at day 182, rats in the TRI group reached a mean weight of 905 ± 108 g, compared to 704 ± 53 g for HF and 594 ± 31 g for ST. These values at the beginning, middle, and end of our timeframe indicate a consistent divergence in body weight among the groups, with rats in the TRI group gaining more weight over time than those in the HF or ST groups. As seen in the graph, CR data support observed ST diet weight data and are used to supplement early growth curves.

**Figure 2:**
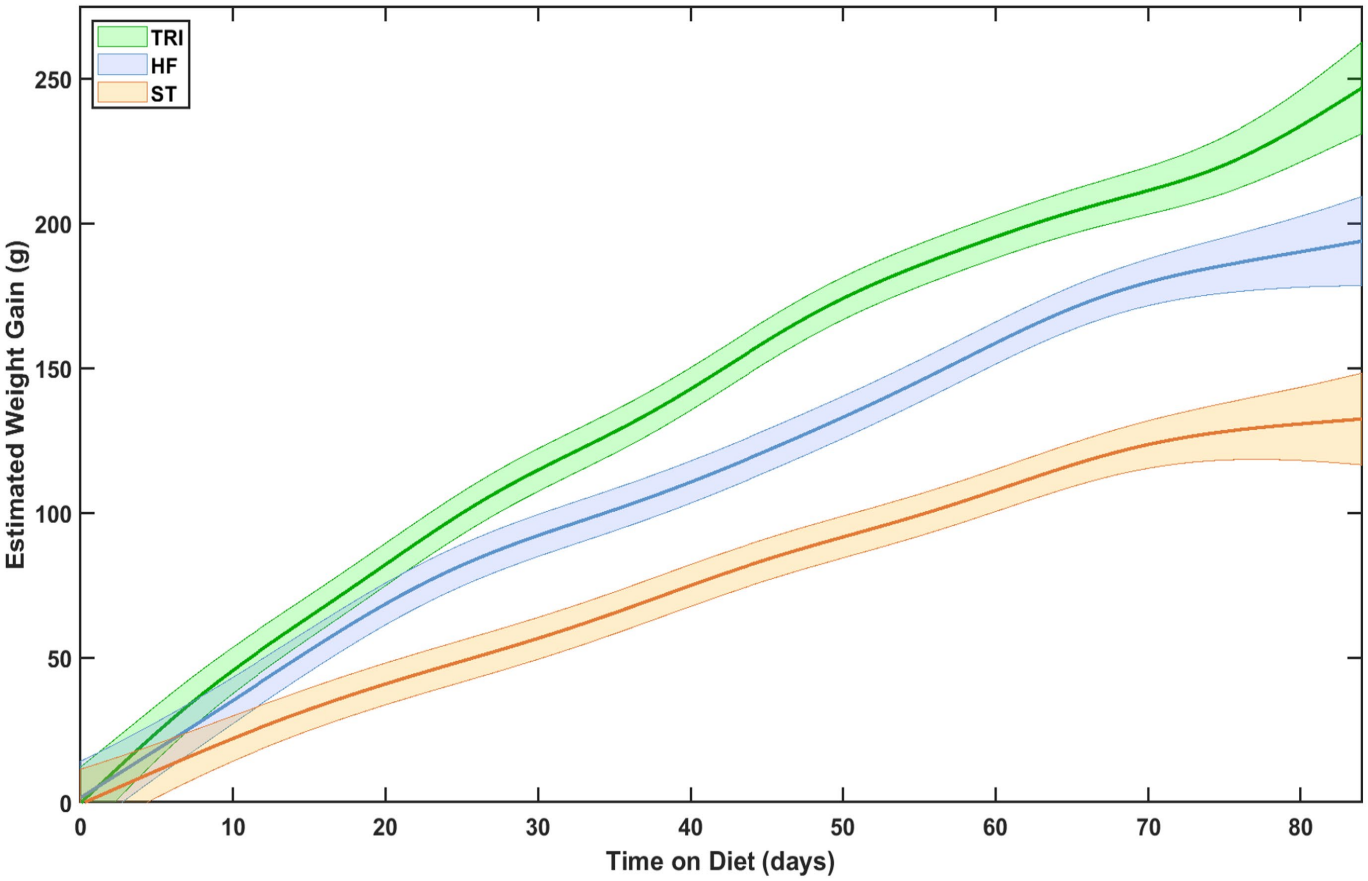
Estimated weight gain as a function of time on diet and diet type. Thick lines are averages, surrounded by a shaded 95% confidence interval.

**Figure 3:**
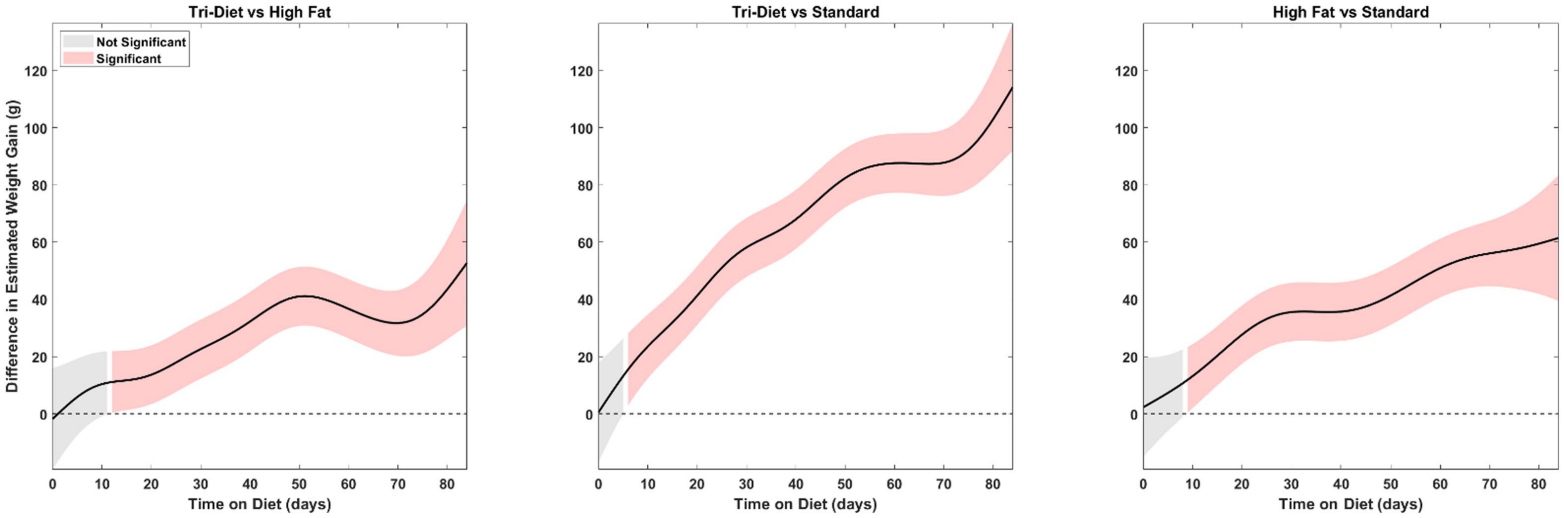
Pairwise difference comparison of diets showing when weight-gain trajectories significantly diverged; shaded regions represent 95% confidence intervals. Pink regions indicate statistically significant divergence (*p* ≤ 0.05) while gray regions indicate that the estimated weight gain is not significantly different between the tested groups.

To evaluate when weight gain diverged among diet groups, we analyzed fitted values from the baseline-adjusted GAMM. Figure 2 shows the estimated weight gain over time for each group, with 95% confidence intervals calculated from the model’s diet-specific smooths. Rats in the TRI group showed divergence from the ST earlier than they did from the HF group. There was no subsequent overlap in confidence intervals between the TRI group and the ST or HF groups after divergence. Throughout the observation period, neither the HF nor the ST groups exhibited growth rates exceeding those of the TRI group. These findings suggest that the TRI diet produced an early and sustained increase in body weight gain distinct from both the HF and ST diets. To formally assess divergence in weight gain, we performed a pairwise difference comparison of diets. The analysis (Figure 3) showed that group differences were statistically significant (*p* ≤ 0.05), with divergence emerging by day 6 for TRI vs. ST, day 9 for HF vs. ST, and day 12 for TRI vs. HF, and remaining significant thereafter.

### Food consumption

**Figure 4:**
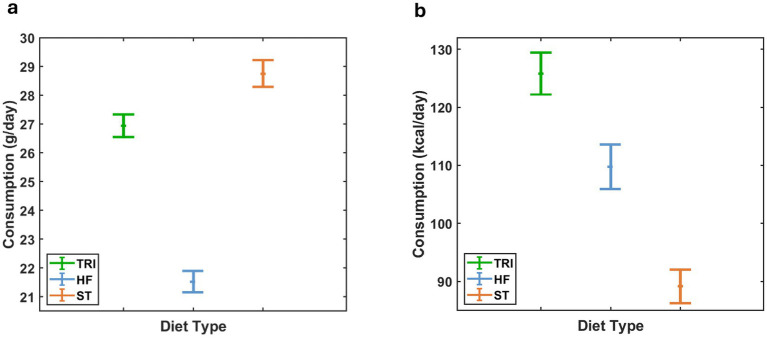
95% confidence interval for the mass of food consumed per day **(a)** and its estimated caloric equivalent **(b)** as a function of diet type. Data revealed that although the ST group consumed the most food per day by mass, it consumed the fewest calories, whereas the TRI group consumed significantly more calories than the HF or ST groups.

From day 98–175, rats in the ST group consumed significantly more food by weight than those in the other groups (*p* < 0.001, *F* (2, 1778) = 330.02). Rats in the TRI group consumed significantly more food by weight than those in the HF group (*p* < 0.001, *F* (1, 1,389) = 375.77). Mean daily food consumption, averaged over the entire timeframe and presented in Figure 4a, was highest in the ST group (28.75 ± 0.47 g), followed closely by TRI (26.95 ± 0.39 g), and lowest in HF (21.53 ± 0.38 g). This pattern was consistent throughout the timeframe.

However, consumed mass is not the sole factor affecting weight gain. By using the energy density for each diet (Table 1), we estimated the equivalent caloric consumption. Throughout the study, rats in the TRI group consumed significantly more calories than those in the HF and ST groups, with the HF group showing higher caloric intake than the ST group (Figure 4b) (*p* < 0.001, *F* (2, 1778) = 291.92). Daily caloric intake averaged across the entire timeframe was 126 ± 2 kcal, 110 ± 2 kcal, and 89 ± 1 kcal for TRI, HF, and ST diets, respectively.

## Discussion

Our findings demonstrate that rats fed the TRI diet gained significantly more weight over the 12-week-period compared to those on the HF and ST diets. By day 182, rats in the TRI group were 28.6% heavier than those in the HF group and 52.4% heavier than those in the ST group. This suggests that TRI rats gain more weight in the same timeframe as rats on single-source diets. Our data suggest that the increased weight gain in the TRI group resulted from greater caloric consumption. TRI rats consumed 14.6 and 41.1% more calories per day than rats on HF or ST diets, respectively. TRI rats demonstrated a preference for but not an exclusive consumption of the HF diet. Among rats in the TRI group, the average composition of food intake by mass over the study period consisted of 75.7% HF, 12.2% HG, and 12.1% ST. However, when comparing the high-fat subcomponent of the TRI consumption with that of the HF group, the TRI group consumed 0.4 g less HF food per day, which was statistically significant (*p* < 0.001) but likely had a negligible contribution to their overall weight gain. Thus, the TRI group consumed nearly the same amount of HF diet as the HF group but consumed additional calories from the HG and ST diets that were not available to the HF group, suggesting that dietary variety promotes overall higher daily caloric intake. However, because direct metabolic measurements to assess diet-induced metabolic changes were beyond the scope of this study, weight gain cannot be definitively attributed to caloric intake alone.

Table 2 summarizes diet formulations, study conditions, and the reported rate of weight gain across several studies. It also contains the probability (p) that the reported rate of weight gain falls within the calculated rate of weight gain for rats on the TRI diet at the same age. In all cases, rats on the TRI diet gained weight at a greater rate than rats in the reported studies. However, outcomes vary widely, even for similar diets, reflecting differences in macronutrient composition, sourcing, and experimental design. This variability has been a persistent challenge for obesity researchers, as it complicates cross-study comparisons and limits reproducibility. The fact that rats in the TRI group reliably outpaced both HF and ST cohorts in terms of estimated weight gain and calorie consumption suggests that dietary variety itself is a critical driver of accelerated weight gain. Our findings position the TRI diet as a robust alternative since the magnitude of weight gain emerged despite variability in animal age and food type preference. Observed macronutrient composition, 19.6% ± 0.84% protein, 26.4% ± 4.62% carbohydrates, and 54% ± 5.01% fat, was determined based on a representative 12-week period of quantified food type consumption (week 14–25) during which there was little variability. Since the rats had *ad libitum* access to three distinct diets instead of a homogenized diet, it is not possible to define the macronutrient contents precisely. At one extreme, a single rat showed a strong preference for HG throughout the study, with corresponding macronutrient percentages of 20.42% protein, 35.74% carbohydrates, and 43.84% fat. These percentages differ from the observed average macronutrient percentages for the TRI group. Nonetheless, this animal achieved a maximum weight of 938 g at the end of the statistical analysis period (week 26), which exceeded the average weight in the TRI group at that time point but was not considered an outlier. Comparatively, a rat with a different preference, favoring the HF food type, 19.69% protein, 23.16% carbohydrates, and 57.15% fat, attained a nearly identical weight of 923 g. This comparison suggests that final weight is consistent despite the differences in dietary preference and variability in macronutrient composition. These findings align with the literature, suggesting that access to multiple palatable options promotes excess body weight due to overconsumption. By producing heavier animal models in a shorter timeframe, the TRI diet offers practical advantages: it reduces study duration and costs while providing a more reproducible approach for inducing obesity.

**Table 2:** A review of selected diets used in obesity studies and their effects on weight gain.

% Calories from			Sugar solution	Rat strain	Study duration (weeks)	Age at start (weeks)	Reported weight gain (g/wk)	TRI weight gain over matched duration and age range (g/wk)*	*p*	Ref
Fat	Carbs	Protein								
Majority of calories from fat										
68	15	17	–	Wistar	17	6	13.28	37.40 ± 4.10	<0.001	(17)
60	20	20	–	SD	8	3	38.75	63.43 ± 5.37	<0.001	(12)*
60	20	20	–	SD	8	8	15	50.17 ± 14.68	0.008	(12)*
50	27	23	–	Wistar	32	3.4	22.66	30.88 ± 2.43	<0.001	(7)*
49	37	14	–	Wister	8	11	12.57	33.47 ± 6.09	<0.001	(19)
45	35	20	–	SD	17	7	16.24	36.50 ± 7.60	0.004	(6)
45	35	20	–	Wistar	17	7	18.66	36.50 ± 7.60	0.009	(6)
Majority of calories from carbohydrates										
10	70	20	–	SD	8	3	34.38	63.43 ± 5.37	<0.001	(12)*
10	70	20	–	SD	8	8	16.4	50.17 ± 14.68	0.011	(12)*
13	67	20	–	SD	17	7	12.28	36.50 ± 7.60	<0.001	(6)
13	67	20	–	Wistar	17	7	11.18	36.50 ± 7.60	<0.001	(6)
15	60	25	–	Wister	8	11	6.646	33.47 ± 6.09	<0.001	(19)
12.4	58.9	28.7	–	Wistar	32	3.4	16.41	30.88 ± 2.43	<0.001	(7)
18	58	24	–	SD	18	6	25.81	36.20 ± 4.65	0.001	(18)
34	49	17	–	Wistar	17	6	14.43	37.40 ± 4.10	<0.001	(17)
17	43	40	–	Wistar	17	6	11.37	37.40 ± 4.10	<0.001	(17)
High fat + water with added sugars										
60	20	20	30% Sucrose	SD	8	3	35	63.43 ± 5.37	<0.001	(12)*
60	20	20	30% Sucrose	SD	8	8	25	50.17 ± 14.68	0.043	(12)*
44.4	40.3	15.3	25% Fructose	Wistar	20	8	24.4	30.65 ± 6.43	0.166	(8)
44.4	40.3	15.3	25% Glucose	Wistar	20	8	29.5	30.65 ± 6.43	0.429	(8)
60	35	5	30% Fructose	Wistar	10	6	7.9	49.86 ± 5.35	<0.001	(13)
80	0	20	37.5% Sucrose	Wistar/Kyoto	5	17	9.2	20.6 ± 4.69	0.008	(14)**

*Tridiet weight gain calculated over an age-aligned interval; adjusted by 1 week when early data were unavailable. **Indicates studies in which animals were provided with a sugar-supplemented drinking solution in addition to untreated drinking water, allowing voluntary consumption.

### Implications in research

Our results suggest that the TRI diet promotes accelerated weight gain in rats, leading to a more rapid development of DIO. Although our data did not extend long enough to determine whether body weight converges across dietary groups, TRI rats consistently gained more weight at a faster rate than single-diet groups. This accelerated DIO can be useful for shorter-duration studies in which rapid weight gain is necessary to evaluate obesity-related outcomes within limited timeframes. For these studies, the TRI diet may improve efficiency by reaching this weight sooner, thereby reducing experimental duration and associated research costs.

Previous studies have shown that supplementing a solid HF diet with sugar water can exacerbate diet-induced obesity, supporting the idea that concurrent access to fat- and carbohydrate-derived calories accelerates weight gain (8, 12–14). However, when outcomes from these additional models are compared with those from the present TRI diet, the magnitude of weight gain remains lower than that observed in our study (see Table 2). Although one study (8) reported similar weight gain that was not significantly less than that of our TRI group, it nevertheless remained lower.

A key distinction between these models lies in dietary choice. In many HF + sugar water studies, animals are provided only sugar water, requiring consumption to maintain hydration. In contrast, animals in the TRI diet paradigm had *ad libitum* access to multiple solid dietary options alongside untreated drinking water and therefore could have consumed fewer carbohydrates by choice. Notably, even when choice is incorporated into sugar-supplemented models, weight gain remains lower than that observed with the TRI diet (14). In a study in which rats were given access to both sucrose-supplemented water and untreated drinking water in combination with an HF diet, animals gained more weight than those fed an HF diet alone, but still significantly less than animals in the TRI diet group (14). Interestingly, this same study reported that, in a prior experiment, the addition of sugar water to an HF diet resulted in reduced weight gain compared to an HF diet alone, highlighting variability across experimental contexts. Taken together, these findings suggest that, while increased caloric availability from combined fat and carbohydrate sources contributes to diet-induced obesity, it does not fully account for the magnitude of weight gain observed in the TRI diet model.

Additionally, because TRI rats achieve significant weight gain at an earlier age, this paradigm could also provide an approach to model childhood obesity. Rates of obesity in children and adolescents have risen sharply worldwide in recent years, raising serious concerns about the long-term health of affected individuals. Early development of obesity has been linked to premature onset of cardiovascular and renal disease, with some projections warning that younger generations may face shorter lifespans than their parents if these trends continue (15). For this reason, developing animal models that capture the onset of obesity during youth is critical for understanding the fundamental pathological changes that occur during this sensitive developmental period. However, most existing preclinical research has focused on adult male animals, leaving a critical gap in models that represent childhood or female obesity (16). By inducing obesity earlier in life, the TRI paradigm may help address this gap when studying early-onset obesity and its long-term health consequences.

### Limitations

The possibility that diet palatability declined over time should be considered, as shifts in consumption may reflect changes in flavor or texture rather than true dietary preference. In our study, all diets were replenished at least every 7 days, with additional food added as needed to ensure *ad libitum* access (10). One concern is the potential for the HF diet to undergo fat oxidation or rancidification during the week. If this occurred, it is conceivable that rats on an HF diet would consume less per day after the food is introduced and that rats on a TRI diet might compensate by increasing their intake of HG chow and ST chow as the week progressed. However, there was no obvious trend when looking at HF consumption throughout the week; therefore, this hypothesis was not supported.

Sample size may be considered a limitation due to the relatively small number of rats per diet group (n = 8–12). However, weekly weight measurements over the 12-week period yielded 427 observations, while daily monitoring of food intake generated 1,795 observations. While smaller group sizes can reduce statistical power, the high-resolution longitudinal data enabled robust statistical modeling, and significant differences emerged between diet groups.

As this study was exploratory rather than a prospectively powered experiment, *a priori* power calculations and a prespecified primary outcome were not defined, and multiple-comparison adjustments were not applied. Although this is an inherent limitation of retrospective analyses, the large number of repeated measurements per rat provided substantial statistical information, enabling precise modeling of growth trajectories and the detection of consistent, biologically meaningful differences across diet groups. Future controlled studies should establish primary endpoints in advance and incorporate appropriate power calculations and multiplicity control.

Another important consideration is the difference in housing and environmental conditions within our analysis and across existing literature. As this was a retrospective study, certain variables, such as water quality, bedding material, time of year, and level of animal interaction, were not measured or fully standardized across studies. Some animals were housed at different facilities, which may introduce minor environmental variation; however, all rats were housed individually and indoors under regulated temperature conditions and maintained on consistent 12-h light/12-h dark cycles, minimizing potential circadian or seasonal effects. These types of differences are common in animal research and do not typically preclude meaningful cross-study comparisons.

Rats in our study were housed in larger cages (approximately 50 × 50 × 50 cm), which could potentially influence activity levels and energy expenditure. However, in studies where cage size was consistent, the TRI group achieved significantly greater weight than their HF and ST counterparts. Rats on a TRI diet also exceeded the weight gain reported in studies in which rats were housed in smaller cages or shared living space (5, 17, 18). While increased cage size might be expected to promote physical activity and reduce weight gain, our findings suggest that dietary composition and variety were the primary drivers of weight differences. Nevertheless, the potential interaction between housing dimensions and dietary interventions warrants further controlled investigation.

Additionally, this was a retrospective analysis of data obtained from other studies. As such, the data are not perfectly aligned in terms of animal age and diet onset as would be expected in a planned, controlled study. ST rats were maintained on their diet since weaning, while TRI and HF rats consumed ST diets before starting their respective diets. With observations starting at day 98, TRI rats had been on their diet for 71 ± 3 days, whereas HF rats were on their diet for 29 ± 7 days (an average of 42 extra days). This difference in time on diet at the start of the analysis likely allowed for increased weight gain for TRI rats since they were placed on an obesity-inducing diet earlier. However, time on the diet did not affect calories consumed or estimated weight gain, which was consistently greater in TRI rats. Furthermore, the heaviest average body weight of the HF group (approximately 850 g on day 280) was exceeded by the average body weight of the TRI group on day 158, 122 days prior, which far exceeds the 42 extra days the TRI rats had been on their diet.

To formally evaluate whether differences in pre-study diet duration biased the post-day-98 results, we fit supplemental generalized additive mixed models (GAMMs) that included both initial weight and pre-study diet duration as covariates. Pre-study diet duration was not a significant predictor of weight gain (*p* = 0.526), and its inclusion did not alter the shape of the growth curves, the relative separation between diet groups, or the estimated days at which diets diverged. Initial weight was significant, but adjusting for it produced nearly identical dietary contrasts and smooth trajectories. Full model outputs are provided in the Supplementary material. Collectively, these analyses indicate that the unequal time on diet prior to day 98 did not affect the conclusions regarding differential weight gain across diets. Food intake measurements were not recorded for the final week (week 26) for the HF group due to scheduling challenges during data collection. These missing data are unlikely to have introduced systematic bias, as HF food consumption over the preceding 8 weeks exhibited minimal variability, as shown in Supplementary Figure 1.

The TRI diet appears to be an efficient model for DIO when rapid weight gain is the primary goal. However, it offers limited control over macronutrient composition because rats choose from three separate food sources rather than a single homogenized pellet. Therefore, if a study requires a precise macronutrient breakdown in addition to promoting weight gain, the TRI diet may not be the most suitable approach.

While these findings strongly suggest that the TRI diet results in heavier rats earlier in a study, future investigations should be conducted under rigorously controlled conditions with all variables being equal except the diet. Additional work can assess how dietary variety, feeding schedules, and macronutrient composition contribute to weight gain. The amount of dietary variability (e.g., three sources vs. four sources or more) could be studied to determine an optimal number and type of sources to achieve DIO within specific time periods. Direct metabolic analyses examining insulin sensitivity, adipokines, and neural reward pathways will be essential to understanding the TRI model’s physiological effects. Finally, adapting the model to reflect human diets or younger cohorts may enhance its translational relevance.

## Conclusion

In this retrospective analysis, the TRI model, which combines HF, HG, and ST diet options, resulted in significantly higher weight gain in rats over a 12-week-period compared to either HF or ST diets alone. The findings suggest that offering a choice including high-caloric options may increase total caloric intake, thereby accelerating weight gain. Rats in the TRI group consistently outpaced both the HF and ST groups in estimated weight gain and caloric consumption, supporting the hypothesis that dietary variety contributes to increased body weight in rat models. These results are significant for optimizing diet protocols in pre-clinical obesity research and suggest that the TRI model could improve experimental efficiency and provide a robust framework for studying obesity and potential interventions.

## Data Availability

The raw data supporting the conclusions of this article will be made available by the authors, without undue reservation.
