# Supplementary material for: Tridiet-enhanced weight gain in Sprague Dawley rats: a retrospective analysis
Source: Front Nutr. 2026 Feb 11;13:1740398. doi: 10.3389/fnut.2026.1740398 (PMC12932429; doi:10.3389/fnut.2026.1740398)
Supplement: Supplementary file 1 [file Table_1.docx]

**Supplement to Rodriguez et al., 2026**


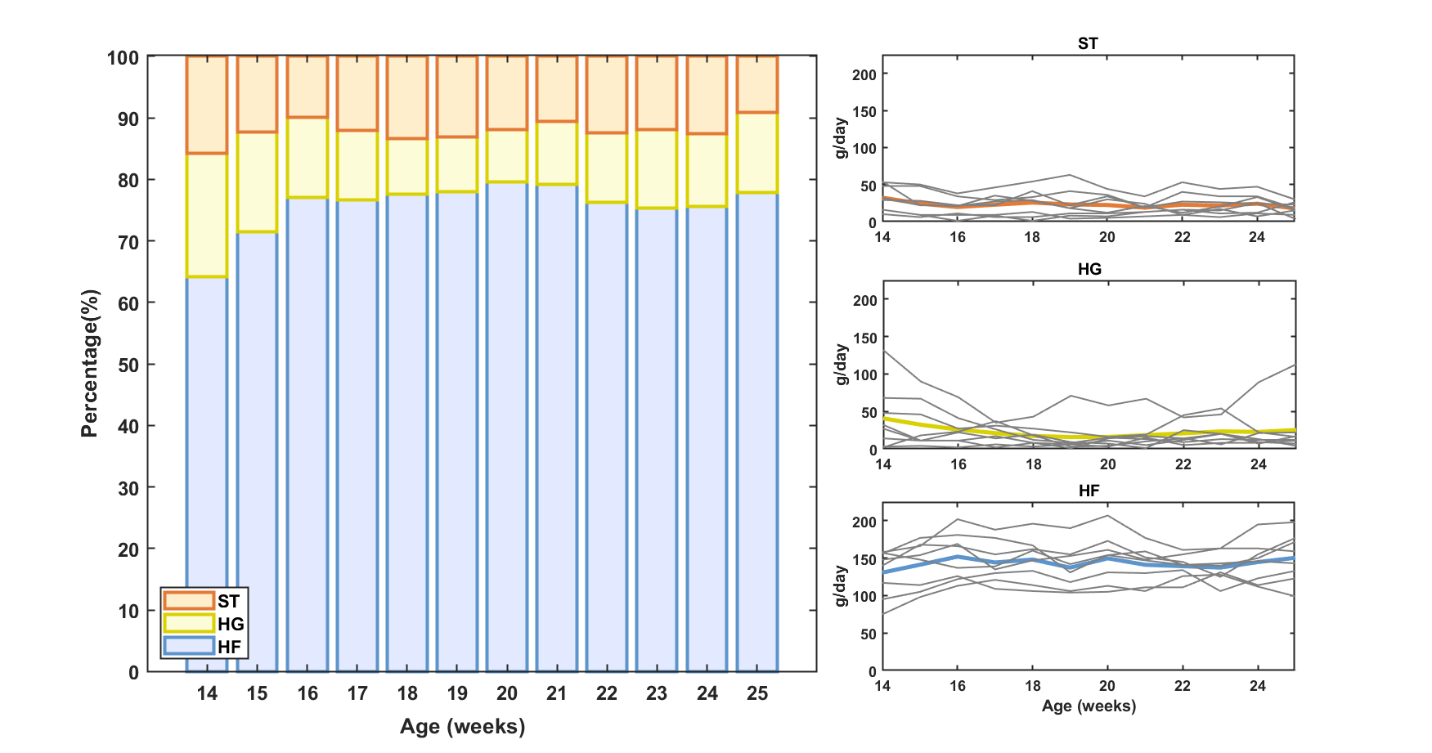


**Figure S1**: Proportion of total grams of food intake by diet type in TRI rats with sub plots of average (colored line) and individual gram consumption data. Within the graphs, orange represents ST sub-diet consumption, yellow represents HG sub diet consumption, and blue represents HF consumption. These graphs display consistent average consumption of each diet sub diet type that stabilizes around week 16.


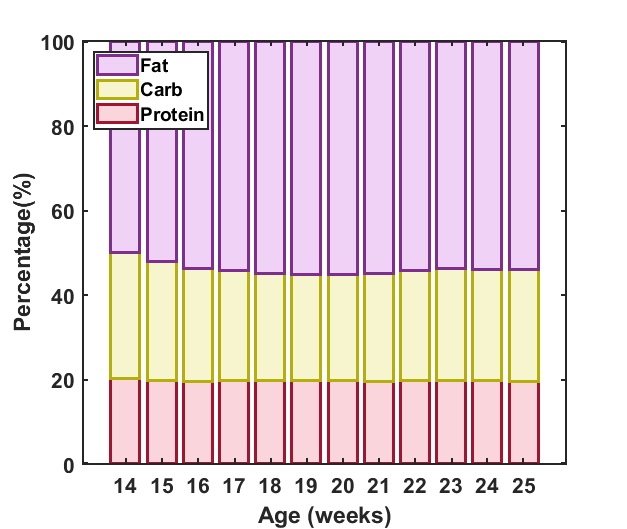

**Figure S2**: When analyzing the proportion of macronutrient sub-type consumption in TRI rats, protein consumption as a percentage of diet remained constant throughout the study while there was a small change in carbohydrate and fat consumption that stabilized by week 16.

**Additional Statistical Analyses**

**Effect of Pre-Study Diet Duration on Weight-Gain Trajectories**

The diet groups differed substantially in their pre-study exposure to their assigned diets prior to formal observation (TRI: 71 ± 3 days, HF: 29 ± 7 days, ST: from weaning). Such imbalance could, in principle, confound post–Day-98 weight-gain trajectories if animals had already undergone metabolic adaptation before the tracking period began. To evaluate whether this pre-existing difference altered our scientific conclusions, we fit a set of generalized additive mixed models (GAMMs) using both weight gain and weight as outcomes. These models sequentially incorporated (1) diet only, (2) diet plus pre-study diet duration and baseline weight, and (3) diet plus baseline weight alone.

**Parametric Effects and Confounding Assessment**

Across all weight-gain models (Table S1), baseline weight was a statistically significant predictor of post–Day-98 growth, while pre-study diet duration was not (p = 0.526 in the fully adjusted model). Diet-group differences remained strongly significant and of similar magnitude across all model specifications. The parallel weight models (Table S5) show the same pattern: baseline weight consistently explains early level differences, whereas pre-study diet duration provides no additional explanatory power.

**Stability of Growth-Curve Shapes**

To assess whether pre-study exposure altered the form of the growth trajectories, we compared effective degrees of freedom (EDF), which tells us how flexible each diet-specific trajectory is (Tables S2 and S6). EDF values were nearly identical across adjusted and unadjusted models. This indicates that the shape of the growth curves does not depend on whether pre-study diet duration or baseline weight is included. Each diet group demonstrates a distinct but stable growth trajectory.

**Model Fit and Robustness**

Model fit statistics (Tables S3 and S7) further support this conclusion. While adding initial weight improves model fit substantially, adding pre-study diet duration yields no meaningful improvement. Importantly, no model shows any reversal or attenuation of diet effects.

**Divergence Timing Between Diet Groups**

Finally, we examined whether adjusting for pre-study exposure changed the estimated days at which diet groups diverged. Table S4 shows that divergence timing is highly stable across all three weight-gain models. The earliest divergence (Tridiet vs Standard) is identical in every specification, and the ordering of all divergences is unchanged. Minor numerical variation appears only in the Tridiet vs HF comparison (Days 12–21) but does not affect interpretation.

Together, these results demonstrate that although the diet groups differed in their pre-study exposure, this imbalance did not meaningfully influence post–Day-98 weight-gain trajectories, did not distort the shape of the growth curves, and did not alter the estimated timing or ordering of divergence between diet groups. The scientific conclusions regarding differential effects of the diets remain robust to all reasonable adjustments.

**Table S1. Parametric Effects: Weight-Gain Models With and Without Adjustment**

| Term | Estimate | Std. Error | t-value | p-value |
| --- | --- | --- | --- | --- |
| Model 1: Diet Only | | | | |
| Intercept | 111.93 | 2.54 | 44.01 | < 0.0001 |
| Diet Standard | -37.17 | 2.89 | -12.85 | < 0.0001 |
| Diet Tridiet | 26.85 | 1.31 | 20.52 | < 0.0001 |
| Model 2: Diet + Pre-diet Time + Baseline Weight | | | | |
| Intercept | 98.36 | 4.87 | 20.19 | < 0.0001 |
| Diet Standard | -39.31 | 3.76 | -10.45 | < 0.0001 |
| Diet Tridiet | 21.46 | 1.62 | 13.25 | < 0.0001 |
| Baseline Weight | 0.0238 | 0.00419 | 5.69 | < 0.0001 |
| Prediet Time | 0.0484 | 0.0762 | 0.63 | 0.526 |
| Model 3: Diet + Baseline Weight | | | | |
| Intercept | 99.60 | 3.41 | 29.17 | < 0.0001 |
| Diet Standard | -35.97 | 2.94 | -12.23 | < 0.0001 |
| Diet Tridiet | 23.43 | 1.39 | 16.80 | < 0.0001 |
| Baseline Weight | 0.0241 | 0.00418 | 5.75 | < 0.0001 |

**Table S2. Growth-Curve Smoothness (EDF) for Weight-Gain Models**

| Model | HF EDF | Standard EDF | Tridiet EDF |
| --- | --- | --- | --- |
| Weight Gain Model 1 | 7.48 | 4.32 | 7.50 |
| Weight Gain Model 2 | 7.48 | 4.29 | 7.51 |
| Weight Gain Model 3 | 7.48 | 4.31 | 7.51 |

**Table S3. Model Fit Summary for Weight-Gain Models**

| Model | Adjusted R^2^ | Scale Estimate |
| --- | --- | --- |
| Weight Gain Model 1 | 0.798 | 76.896 |
| Weight Gain Model 2 | 0.802 | 76.894 |
| Weight Gain Model 3 | 0.801 | 76.895 |

**Table S4. Estimated Days of Divergence Between Diet Groups**

| Comparison | Weight Gain Model 1 | Weight Gain Model 2 | Weight Gain Model 3 |
| --- | --- | --- | --- |
| Tridiet vs Standard | Day 5 | Day 5 | Day 5 |
| Tridiet vs HF | Day 12 | Day 21 | Day 13 |
| HF vs Standard | Day 9 | Day 12 | Day 9 |

**Table S5. Parametric Effects for Weight Models**

| **Term** | **Estimate** | **Std. Error** | **t-value** | **p-value** |
| --- | --- | --- | --- | --- |
| **Model W1: Weight ~ Diet + s(Days_on_Diet)** | | | | |
| Intercept | 638.98 | 2.37 | 269.92 | < 0.0001 |
| Standard vs HF | -98.18 | 2.76 | -35.64 | < 0.0001 |
| Tridiet vs HF | 172.12 | 1.49 | 115.45 | < 0.0001 |
| **Model W2: Weight ~ Diet + Baseline Weight + Prediet Time + s(Days_on_Diet)** | | | | |
| Intercept | 88.16 | 3.03 | 29.08 | < 0.0001 |
| Standard vs HF | -42.21 | 2.94 | -14.37 | < 0.0001 |
| Tridiet vs HF | 19.12 | 1.68 | 11.40 | < 0.0001 |
| Initial Weight | 1.052 | 0.0046 | 227.10 | < 0.0001 |
| Prediet Time | 0.048 | 0.046 | 1.05 | 0.294 |
| **Model W3: Weight ~ Diet + Baseline Weight + s(Days_on_Diet)** | | | | |
| Intercept | 90.16 | 2.67 | 33.77 | < 0.0001 |
| Standard vs HF | -39.01 | 1.58 | -24.76 | < 0.0001 |
| Tridiet vs HF | 21.28 | 1.19 | 17.95 | < 0.0001 |
| Initial Weight | 1.050 | 0.0046 | 227.48 | < 0.0001 |

**Table S6. Smooth-Term Flexibility (EDF) for Weight Models W1–W3**

| Model | HF EDF | Standard EDF | Tridiet EDF |
| --- | --- | --- | --- |
| Model W1 | 6.02 | 3.82 | 5.35 |
| Model W2 | 6.12 | 4.09 | 5.84 |
| Model W3 | 6.14 | 4.12 | 5.83 |

**Table S7. Model Fit Summary for Weight Models W1–W3**

| Model | Adjusted R^2^ | Scale Estimate |
| --- | --- | --- |
| Model W1 | 0.798 | 0.000296 |
| Model W2 | 0.944 | 0.000247 |
| Model W3 | 0.944 | 0.000247 |

**Comparison of GAMM and Linear Mixed-Effects Models**

A comparison between generalized additive mixed models (GAMMs) and simpler linear mixed-effects models (LMMs) was conducted to determine whether the nonlinear trajectories estimated by the GAMM were necessary and whether the additional model flexibility was supported by the data. The LMM assumes strictly linear growth patterns and linear diet-by-time interactions, whereas the GAMM permits nonlinear diet-specific curves that may better represent biological weight trajectories. This section summarizes differences in model fit, predictive accuracy, and spline diagnostics to evaluate the appropriateness of using a GAMM for the primary analysis.

Model adequacy was first assessed using the Akaike Information Criterion (AIC), which balances goodness-of-fit against model complexity. The GAMM showed a substantially lower AIC than the LMM, indicating better fit even after penalizing for additional flexibility.

**Table S8. AIC Comparison Between GAMM and Linear Mixed Model**

| Model | AIC |
| --- | --- |
| GAMM (Model W1) | 17,730.46 |
| Linear Mixed Model | 22,559.81 |

To assess generalizability, 5-fold cross-validation was performed with folds stratified by Rat ID. Mean squared error (MSE) was calculated on held-out data for each fold. The GAMM achieved lower predictive error than the LMM, confirming that the nonparametric structure enhances rather than compromises prediction accuracy.

**Table S9. Five-Fold Cross-Validated Prediction Error**

| Model | Mean MSE |
| --- | --- |
| GAMM (Model W1) | 3,433.5 |
| Linear Mixed Model | 3,498.1 |

Spline behavior was evaluated using the effective degrees of freedom (EDF), the k-index, and concurvity. EDF reflects the flexibility of each smooth term; values that are moderate rather than extremely high suggest appropriate curvature. The k-index tests whether the chosen spline basis dimension is adequate; values near 1.0 indicate correct specification. Concurvity quantifies redundancy between smooth terms; values near zero imply that the smooths are distinct and not approximating one another. The EDF values demonstrate that each diet exhibits its own trajectory without excessive wiggliness. The k-index values of 1.0368 indicate that the basis dimensions were adequate for representing each curve.

**Table S10. EDF and k-index for Diet-Specific Smooths**

| Smooth Term | k' | EDF | k-index | p-value |
| --- | --- | --- | --- | --- |
| s(Days_on_Diet): HF | 9 | 6.02 | 1.0368 | 1.00 |
| s(Days_on_Diet): Standard | 9 | 3.82 | 1.0368 | 1.00 |
| s(Days_on_Diet): Tridiet | 9 | 5.35 | 1.0368 | 1.00 |

Concurvity values were uniformly low across all smooth terms, indicating minimal redundancy and confirming that each diet contributes a unique nonlinear pattern rather than correlated smooths.

**Table S11. Concurvity Diagnostics for GAMM Smooth Terms by Diet Group**

| Measure | para | HF | Standard | Tridiet |
| --- | --- | --- | --- | --- |
| Worst | 0.598 | 0.00446 | 0.00159 | 0.00159 |
| Observed | 0.598 | 0.00120 | 0.00052 | 0.00052 |
| Estimate | 0.598 | 0.00156 | 0.00067 | 0.00067 |

Across all evaluation metrics, the GAMM outperformed the linear mixed model. It showed substantially better fit as reflected by lower AIC, yielded improved predictive accuracy in cross-validation, and exhibited well-behaved smooth terms supported by the EDF, k-index, and concurvity diagnostics. Taken together, these results indicate that nonlinear, diet-specific trajectories are a fundamental characteristic of the data, and they justify the use of a GAMM rather than a linear mixed-effects model.
